# A Method for Remotely Sensing Vital Signs of Human Subjects Outdoors

**DOI:** 10.3390/s150714830

**Published:** 2015-06-24

**Authors:** Chuantao Li, Fuming Chen, Jingxi Jin, Hao Lv, Sheng Li, Guohua Lu, Jianqi Wang

**Affiliations:** 1 Department of Biomedical Engineering, Fourth Military Medical University, Xi’an 710032, China; E-Mails: lichuantao614@126.com (C.L.); cfm5762@126.com (F.C.); fmmujxj@fmmu.edu.cn (J.J.); lvhao@126.com (H.L.); sheng@mail.xjtu.edu.cn (S.L.); 2 Shaanxi University of Technology, Hanzhong 723001, China

**Keywords:** 24 GHz Doppler radar, searching for survivors, respiration, moving object interference

## Abstract

After chemical or nuclear leakage or explosions, finding survivors is a huge challenge. Although human bodies can be found by smart vehicles and drones equipped with cameras, it is difficult to verify if the person is alive or dead this way. This paper describes a continuous wave radar sensor for remotely sensing the vital signs of human subjects. Firstly, a compact and portable 24 GHz Doppler radar system is designed to conduct non-contact detection of respiration signal. Secondly, in order to improve the quality of the respiration signals, the self-correlation and adaptive line enhancer (ALE) methods are proposed to minimize the interferences of any moving objects around the human subject. Finally, the detection capabilities of the radar system and the signal processing method are verified through experiments which show that human respiration signals can be extracted when the subject is 7 m away outdoors. The method provided in this paper will be a promising way to search for human subjects outdoors.

## 1. Introduction

Chemical agents and nuclear radiation causing long-term health hazards can potentially be utilized by terrorists [1]. On 11 March 2011, a massive earthquake and tsunami hit eastern Japan, particularly affecting the Tohoku Sea, and causing heavy casualties, property losses, and a major nuclear leakage incident at the Fukushima nuclear plant. In the event of a nuclear or chemical terrorist attack or a nuclear leakage, the urgent task on hand is to search for survivors. However, this is a dangerous situation because of potential exposure of the search teams to toxic chemicals or high radiation levels. Although teleoperated robots can check for nuclear radiation, detect chemicals and find human bodies, it is still difficult to judge whether individuals are alive or not [2]. If the rescuers know who is alive in advance, the rescue will be more efficient and more lives can be saved. Thermal cameras can judge whether the human body has vital signs, but they are expensive and suffer easy interference from the environment. To address this issue in this paper a new small compact and compatible sensor for remotely sensing vital signs is presented.

Based on the Doppler principle, continuous wave (CW) radar monitors the body’s chest movement caused by respiration to estimate the respiration rate [3,4,5,6]. One reason for gathering physiological parameters at a distance in a non-contacting and non-invasive way is that the technique can be applied to medical treatment [7], safety, lie detection, the military field and searching for survivors. In the medical field, contact electrodes cannot be used for detecting the respiratory signals of seriously burnt patients; detection in a non-contacting and non-invasive way using CW radar can completely solve this problem. In addition, more non-intrusive and non-invasive equipment for measuring physiological parameters is required for home healthcare too [8]. In the military field, continuous wave radar can penetrate walls to detect the enemy behind. To avoid the subject knowing he is under lie detection testing, a good method is to detect the physiological parameters by a continuous wave radar sensor while the subject is answering questions [9]. At present, continuous wave radar is mainly used indoors, where there is low interference and the radar directly faces towards the abdomen of human body closely. When the radar works outdoors, there will be great interference caused by many moving objects and the continuous wave radar cannot approach the human body closely due to the complex situation, so the radar system must have anti-jamming performance and remote human respiration detection capabilities. When using continuous wave radar for detecting whether vital signs exist outdoors in a certain area, it is necessary to take the strong interferences from the surroundings into consideration, such as the movement of flowers, grass and leaves caused by the blowing wind. The breathing displacement of the human body is intrinsically relatively small, ranging from 4 to 12 mm [10], and the displacement will be even smaller if radar does not directly face towards the breathing part of the human body. Therefore, it will be more difficult to extract respiration signals from complicated radar echo signals. Facing a new application and new problems, this paper designs a kind of compact and portable CW radar system for detecting the respiration signals of the human body in a non-contacting way at a long distance.

## 2. Description of the 24 GHz Doppler Radar Sensor

In a CW radar system, a sine wave is transmitted to a target where it is reflected. The back-reflected wave reaches the receiving antenna with a time difference depending on the distance to the reflecting target, *i.e.*, the phase difference between transmitted and received signals is directly proportional to the target motion. The transmitted signal sent out by transmitting antenna can be described as below:

(1) $$S_{s}(t)=A\sin(2\pi f_{0}t+\theta_{0})$$

where θ*_0_* is the phase at time *t* = 0, *A* is the amplitude and *f_0_* is the frequency of the transmit signal. The reflected signal can be described as below [11]:

(2) $$S_{r}(t)=KA\sin\left[2\pi f_{0}t+\theta_{0}+2\pi \frac{2x(t)}{\lambda }\;\right]\text{+}n(t)\;$$

where λ is the wavelength of the transmitted signal, signal *x*(*t*) is the variation with time of the distance between radar and target, *n*(*t*) is the noise, that includes electronic circuit and ambient noises. When the system is used for detecting human breathing, the change of *x*(*t*) with time is mainly caused by the human body breathing. Parameter *K* is the attenuation suffered by signal and caused by radar misalignment and free space attenuation. After combining *S_r_*(*t*) with *S_s_*(*t*), we get *S_A_*(*t*) as below:

(3) $$\begin{array}{l} S_{A}(t)=S_{r}(t)•S_{s}(t)\\ \;=\frac{KA^{2}}{2}\left[\cos(2\pi f_{0}t+\theta_{0}+2\pi f_{0}t+\theta_{0}+2\pi \frac{2x(t)}{\lambda })\right]+\frac{KA^{2}}{2}\left[\cos(\theta_{0}-2\pi \frac{2x(t)}{\lambda })\right]+n(t)•A\sin(2\pi f_{0}t+\theta_{0}) \end{array}$$

**Figure 1:**
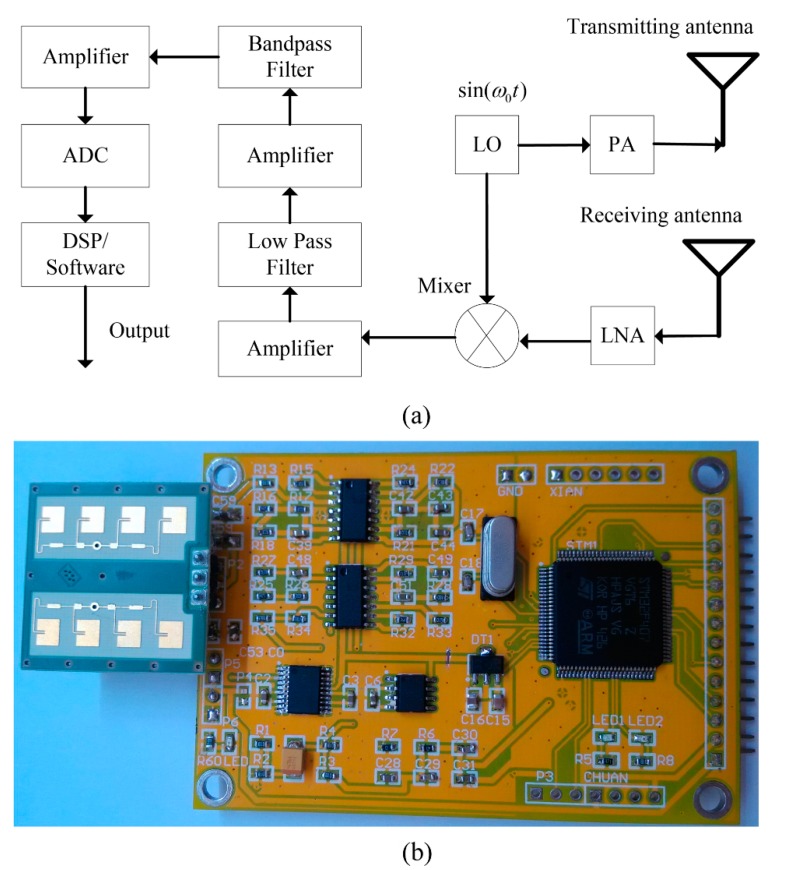
CW radar sensor system. (**a**) Block diagram of the 24 GHz radar respiration sensor system; (**b**) Photograph of the 24 GHz respiration radar sensor system.

A low-pass filter can be used for eliminating the 24 GHz carrier signal. In the low-frequency parts, it is mainly the breathing *x*(*t*) that causes the change of phase position. A low-frequency radar produces in a small phase variation while a high-frequency radar results in a large phase variation. High frequency can reduce the radar antenna size, but high radar frequency leads to strong directivity, which makes the radar alignment difficult during the breathing measurements [12]. Considering the precision and detection scope comprehensively, the 24 GHz radar is selected as shown in Figure 1. The wave length of a 24 GHz radar is 12.5 mm, and the radar can cover 60° in the vertical direction, with a horizontal defensive line covering 160°. It is reported that the chest displacement caused by breathing can result in a 230.4°–691.2° phase change of the 24 GHz continuous radar signal.

It is well known that a single-channel radar has a null detection point which occurs every λ/4. The quadrature radar with I/Q channel can eliminate the null detection problem, but due to radar hardware limitations, the 24 GHz radar is a single-channel radar where a null detection point exists every 3.125 mm. Since the distance difference between a radar signal of movement caused by respiration is longer than 3.125 mm, the null detection point is not a serious problem for a 24 GHz radar system.

As shown in Figure 1a, on the transmitting side, a local oscillator (LO) generates sin(w_0_t) signal, a power amplifier (PA) radiates the sin(w_0_t) signal emitted by the transmitting antenna. On the receiving side, the reflected signals are captured by the receiving antenna, followed by a low-noise amplifier (LNA) and a mixer. The reliable distance for detecting human respiration is about 7 m. After the analog circuits filter and amplifier processing, the STM32F407 uses on-chip 12 bits AD for transforming the returned radar signal from analog to digital. The STM32F407 integrates multiple channels of on-chip 12 bits AD and digital signal process (DSP) core. Figure 1b shows a photograph of the proposed 24 GHz respiration radar sensor hardware system.

## 3. Signal Recording and Processing

The SNR of the signal collected by radar is high indoors when the radar is close to the detected object and directly facing towards the chest and abdomen of the detected object. The frequency of respiration signals under such an ideal situation can be calculated easily. Since there is a breeze, and movement of leaves, flowers and grass during the outdoors experiments, it is difficult to judge whether there is respiration signal in the radar echo. Traditionally, finite impulse response (FIR) or infinite impulse response (IIR) filters can be used for cancelling noise, but the respiration frequency ranges from 0.1–0.65 Hz which overlaps with the noise frequency, so this approach will fail.

Since traditional FIR or IIR filters are insufficient and Fourier analysis cannot work under high noise and moving object interference conditions [11,12], some new signal processing methods are needed to extract respiration signals from mixed signals. Currently, respiration signal extraction from low SNR radar signals is a hot issue. The works described in [11,13,14] adopt different methods to eliminate background clutter and the influence of target body movement. In this paper, body movement is not the biggest problem, as it is easy to judge whether the human is alive or not by camera. What’s more, movement of the target body or a moving person beside the target body only occasionally influence respiration detection; however, wind blowing outdoors generates serious continuous influences which cannot be removed by an influence threshold. This paper focuses on the continuously moving interference of the environment.

**Figure 2:**
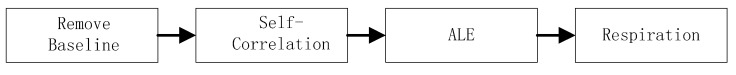
Signal processing block diagram.

The largest difference between respiration signal and noise is periodicity. At present, there are many methods of extracting periodic signal from noise, such as self-correlation, adaptive line enhancer (ALE) [15], blind source separation (BBS) [16], empirical mode decomposition (EMD) [17], *etc.*, nonetheless, the effect is not obvious when only using one of the methods mentioned above. Through research and comparison among these methods, this paper adopts a series of signal processing methods, as shown in Figure 2. Firstly, the radar signal is preprocessed to remove the baseline and background clutter, so as to improve the SNR of respiration signals. Secondly, self-correlation is adopted to improve the respiration signal and reduce colored Gaussian noise signals. Lastly, the ALE method is used for even reducing the Gaussian noise signal of the detected respiration.

### 3.1. Removal of Baseline and Clutter Reduction

In this application, the human body is presumed sedentary. In order to remove the baseline drift of signal and background noise [15], a single-side slip averaging method is adopted, as shown in the following formula:

(4) $$y(n)=S_{A}(n)-\frac{\sum_{j=n-M}^{n}S_{A}(j)}{M+1}$$

where *S_A_*(*n*) is the original signal converted by AD. During baseline elimination, the value of M is important, and the different values of M may greatly influence the baseline elimination. In order to get good quality baseline removal, Equation (4) conducts a Z-transform and the obtained system function of this operation is given by the following formula:

(5) $$H(z)=\frac{Y(z)}{S_{A}(z)}=\frac{-Mz+(M+1)-z^{-M}}{-(M+1)z+(M+1)}$$

**Figure 3:**
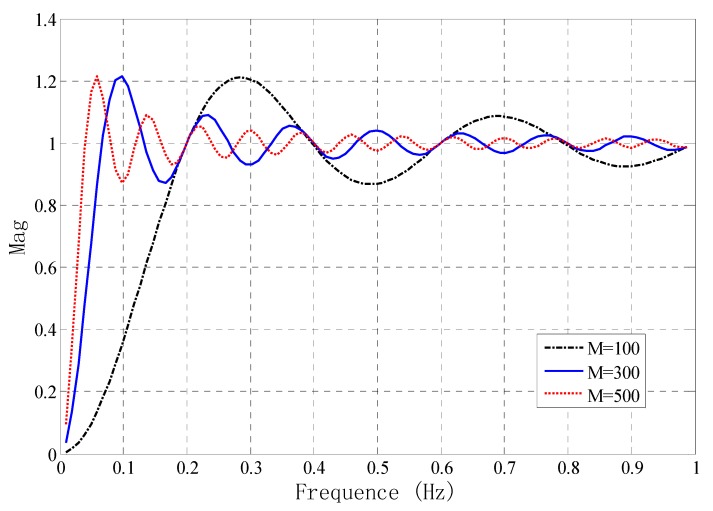
The frequency response of the system for different filter orders.

By changing *M* we solve the frequency response *H*(*z*) in Equation (5). As shown in Figure 3, when *M* equals 100, the fluctuation of the system frequency response is relatively large, and the cut-off frequency is about 0.18, which will weaken some respiration signals. When *M* equals 500, the fluctuation of system frequency response is relatively small after the cut-off frequency, but too long an *M* will seriously delay the signal. When *M* equals 300 (about 7.5 s of window duration), the cut-off frequency is about 0.06 (respiration rate ranges from 0.1 to 0.6); in addition, the amplitude fluctuation of the frequency response after the cut-off frequency is also relatively small.

### 3.2. Signal Self-Correlation

**Figure 4:**
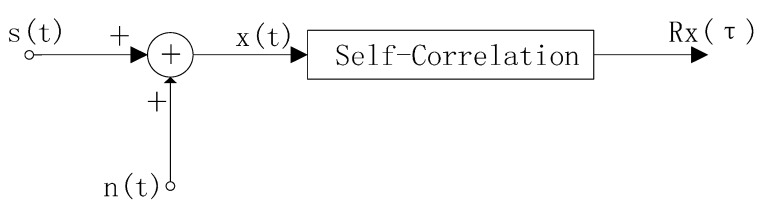
Using self-correlation to extract periodic signal from noise.

The model of using a self-correlation method for recovering useful signals from noise is shown in Figure 4, where *s*(*t*) refers to a useful respiration signal, *n*(*t*) refers to the noise signal which is irrelevant to *s*(*t*), and the signal observed by radar is *x*(*t*):

(6) x(t)=s(t)+n(t)

After self-correlation *x*(*t*) is determined as follows:

(7) $$\begin{array}{l} R_{x}(\tau )=E[x(t)x(t-\tau )]=E\left\{\left[s(t)+n(t)][s(t-\tau )+n(t-\tau )\right]\right\}\\ \;=E[s(t)s(t-\tau )]+E[n(t)n(t-\tau )]+E[s(t)n(t-\tau )]+E[n(t)s(t-\tau )]\\ \;=R_{s}(\tau )+R_{n}(\tau )+R_{sn}(\tau )+R_{ns}(\tau ) \end{array}$$

When *s*(*t*) and *n*(*t*) are irrelevant, *R_sn_*(τ) and *R_ns_*(τ) all equal zero, *n*(*t*) refers to a non-periodic and narrow band signal, which is mainly centered near τ = 0, when τ is relatively large, only the of *R_s_*(τ) will be reflected by *R_x_*(τ). If *s*(*t*) refers to a periodic function, *R_s_*(τ) still refers to the periodic function, with a frequency the same as that of *s*(*t*). In this case, when the τ is relatively large, *R_x_*(τ) can be used for calculating the frequency of *s*(*t*).

### 3.3. Adaptive Linear Enhancement

**Figure 5:**
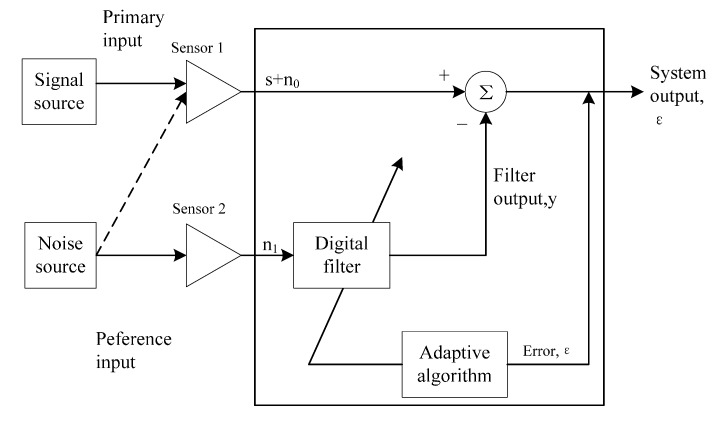
Block diagram of an adaptive noise canceller.

After self-correlation, the SNR of signal is enhanced. However, for a signal with extremely low SNR, it is necessary to further enhance the SNR. The term adaptive cancellation refers to an adaptive filter configuration which is satisfactory in an environment where complete knowledge of relevant signal characteristics is not available. Figure 5 shows the block diagram of adaptive cancellation.

The noise canceller has two inputs, the primary and the reference inputs; and an output, the system output. A sensor is mainly used for receiving signal s, where s is the wanted signal, with the mixed noise n_0_, and another sensor is mainly used for receiving noise n_1_, wherein n_0_ and n_1_ are correlated, while s and n_0_ are not correlated. Through an adaptive algorithm, we can work out the filter coefficient based on the principle of minimum mean square error. Based on the digital filter of this filter coefficient, we can use n_1_ for working out n_0_, and finally, use s + n_0_ of Sensor 1 for deducting the n_0_ output by the digital filter, thus outputting a pure signal s from the system output and playing the functions of filtering and enhancing SNR. For a more detailed treatment of adaptive noise cancellation, the reader is referred to [10].

**Figure 6:**
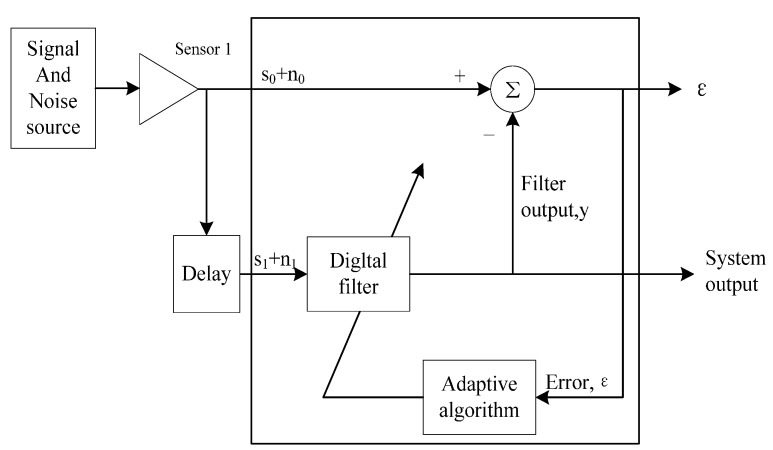
Block diagram of Adaptive Line Enhancement.

Although the adaptive noise canceller is very useful, it is very difficult to use two radars during actual operation, with one radar only collecting noise and the other one collecting both noise and signal, for both noise and signal may come from the same direction, so the sensor cannot distinguish which is noise and which is signal. In order to solve this problem, ALE is adopted. ALE does not need a separate reference signal, which can be obtained by constant delay of the input signal. Figure 6 shows the block diagram of adaptive cancellation.

As shown in Figure 6, s_0_ is a periodic signal and n_0_ is a noise signal. s_1_ + n_1_ is the signal after the time delay of s_0_ + n_0_. After the time delay, s_1_ and s_0_ are still correlated. The n_0_ signal is a non-periodic signal, and after the time delay, it is not related to n_1_. Therefore, the digital filter will output s_1_ which is correlated with s_1_ + n_1_. If the periodic signal refers to the useful signal, which is the same as the respiration signal here, the system output can be output as the desired signal. If the aim is to eliminate the periodic signal from s_0_ + n_0_, ε can be output as the desired signal.

When the primary inputs and reference inputs are determined, many different adaptive filter algorithms can be used, such as last mean squares (LMS), normalized last mean squares (NLMS), and recursive least squares (RLS). From the robustness and convergence rate point of view, NLMS was selected finally. The filter order M was set as 300, and the time delay as 50.

## 4. Results and Discussion

In order to investigate typical situations through a long-time monitoring, we performed measurements on two male volunteers (one 26 years old and the other 28 years old) who participated in the simulation of several positions and cases. This paper mainly refers to three experiments. The first experiment is to verify that the radar has high human body respiration detection accuracy. The second experiment is to check the human respiration sensing ability of the radar system at a long distance. The last one is to prove that the human body respiration signals can be detected by the CW radar system outdoors with continuous serious interference from moving objects.

### 4.1. Indoor Experiments

In order to evaluate the performance of the radar system, we apply a respiratory belt transducer (RBT, MLT1132, AD Instruments Corp, Colorado Springs, CO, USA), which contains a piezoelectric sensor that responds linearly to changes in length and measures the changes of thoracic or abdominal circumference during respiration and is used in respiration monitoring to monitor the respiratory motion of subjects as a referential method. Since the data measured by the two methods differs in scale, so we only focus on the respiration frequency.

**Figure 7:**
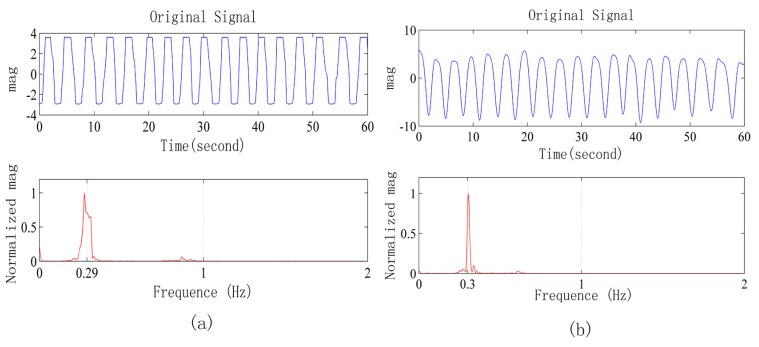
(**a**) Respiration waveforms obtained from the radar and its frequency spectrum; (**b**) Respiration waveforms obtained from the respiratory belt and its frequency spectrum.

The first indoor experiment scenario was described next. The subjects wearing the RBT lie on a cushion, and the radar is 1 m away from their body. We place the radar on the ground, at the same height as the abdomen of the human body, and make the radar antenna directly face the human body. Respiration waveforms measured by the radar system and frequency spectrum are shown in Figure 7a. The respiration waveforms measured by the radar system and frequency spectrum are shown in Figure 7b. Prominent peaks of the two spectra are the same under the frequency accuracy of 2048 Fast Fourier Transform points with 40 Hz sample frequency.

The second indoor experiment scenario was as follows. A person lies on a cushion, and the radar is 7 m away from the human body. We place the radar on the ground, at the same height as the abdomen of the human body, and make the radar antenna directly face the human body. The object to be observed is then tested in four common postures, including lying on his back, lying on his stomach, lying on his side with the back towards the radar, and lying on his side and facing towards the radar.

**Figure 8:**
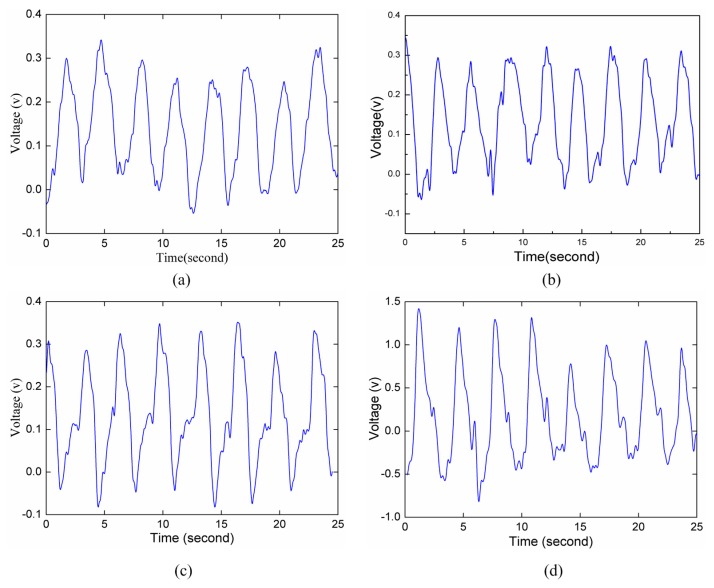
Respiration waveforms detected at different human body postures. (**a**) Lying on his back; (**b**) Lying on his stomach; (**c**) Lying on his side with the back towards the radar; (**d**) Lying on his side and facing towards the radar.

Under such an ideal indoor situation, the respiration waveform can be clearly seen. Especially in Figure 8a–c, the signal amplitudes are basically equal to each other, and the reason is that the distance between human body and radar is unchanged basically in every measurement, and for each time, the measured parameter is the respiration movement of the subject’s ventral abdomen. Figure 8d collects the respiration displacement of the abdomen of the human body, and the signal strength is greater obviously than that in the previous three situations; the reason is that the displacement of abdomen during the respiration movement is large and the movement area is big when the radar directly faces the abdomen.

### 4.2. Outdoor Experiments

The conclusion of the indoor experiments simplified the design of the outdoor experiments, *i.e.*, a person lies on the grass while wind is blowing continuously. The radar was at the same height as the abdomen of the human body, which is within the area covered by the main lobe of the radar, and 3 m, 5 m and 7 m away from the radar, respectively. The object breathes calmly. During the experiment, there is serious interference caused by the breeze, and under such conditions, the respiration signals cannot be directly observed from the collected radar signal waveforms. In order to obtain the respiration frequency exactly and reliably, the experiment time is relatively long, *i.e.*, 3 min for each time.

**Figure 9:**
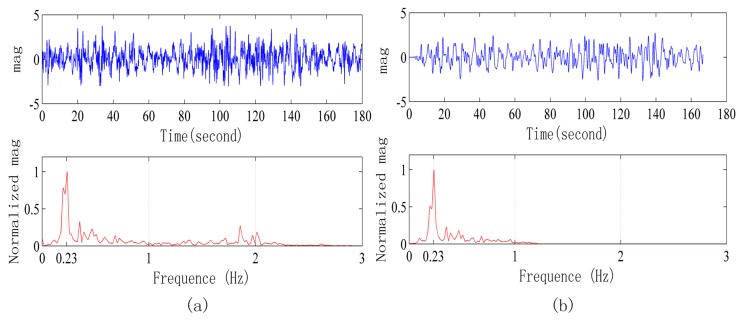
Signals collected in a strong interference environment when the radar is 3 m away from the human body. (**a**) Original signal waveform and frequency spectrum; (**b**) Signal after FIR filter processing and its frequency spectrum.

Figure 9 shows the signals collected in a strong interference environment when the radar is 3 m away from human body. Figure 9 refers to the original signal waveform and its frequency spectrum, and for the frequency spectrum, the improved Welch method is adopted, with overlap of 1024 points, rectangular window and 2048 Fast Fourier Transform points. Figure 9b refers to the signal after applying the low pass FIR filter to Figure 9a and its frequency spectrum. FIR adopts a Hamming window, with filter order of 100, and 1 Hz cut-off frequency. After only FIR filtering, it is still difficult to see s stable and regular respiration waveform in the waveform as shown in Figure 9b.

**Figure 10:**
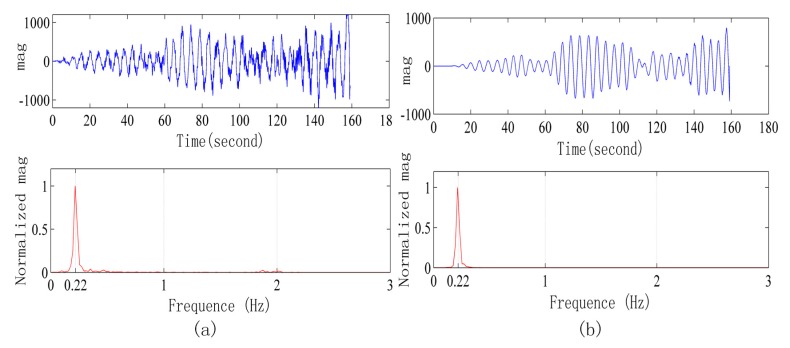
(**a**) Signal waveform of Figure 9a after removing the baseline, self-correlation processing and its power spectrum. (**b**) Signal waveform of Figure 10a after ALE processing and its power spectrum.

Figure 10a refers to the signal after removing the baseline and self-correlation of Figure 9a, taking half of the points and removing 200 points near zero. In order to guarantee the following ALE continuity, the peak value at the zero point is avoided. At this time, the periodic signal of Figure 10a can be seen; however, there is a lot of noise at the same time. Then, we conduct processing of the signal in Figure 10a by the ALE method, and the signal after processing is shown in Figure 10b, where the periodic signal is clear.

**Figure 11:**
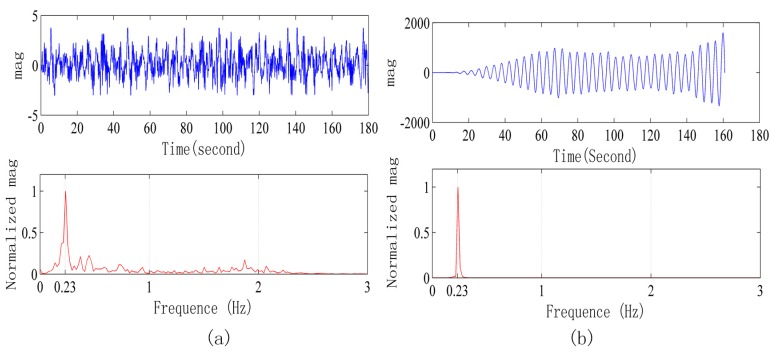
Signals waveform collected in a strong interference environment when the radar is 5 m away from human body. (**a**) Original signal waveform and its frequency spectrum; (**b**) Signal waveform after processing and its power spectrum.

**Figure 12:**
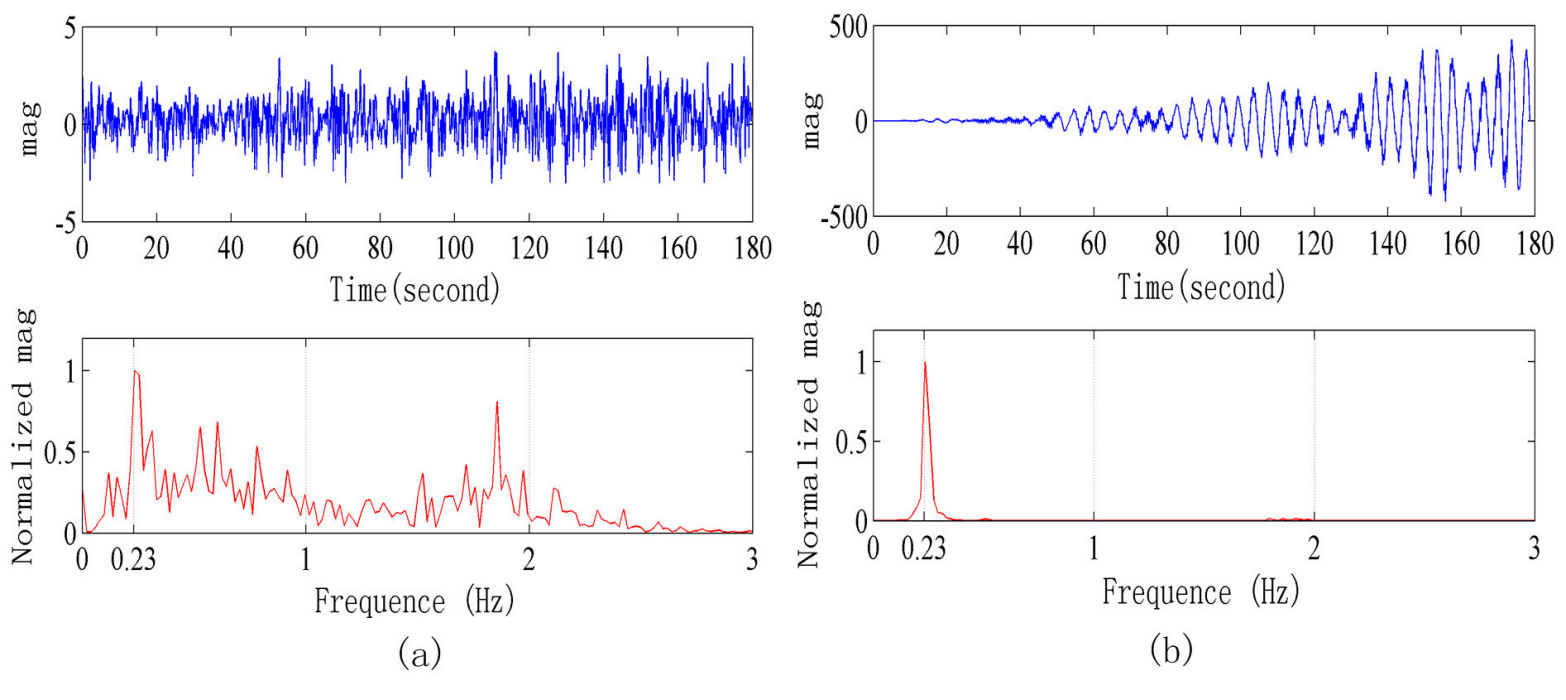
Signals collected in a strong interference environment when the radar is 7 m away from the human body. (**a**) Original signal waveform and its frequency spectrum; (**b**) Signal after the processing and its power spectrum.

Figure 11 and Figure 12 are the signals when the radar is 5 m and 7 m away from the human body, respectively. After adopting the signal processing methods, the respiration signal waveform and exact respiration frequency can be obtained.

For the quantitative analysis, the SNR of the respiration signal is redefined in the frequency domain [14]. The breathing rate is estimated as the frequency *f_max_* of the peak in the frequency spectrum. The SNR before processing is calculated as below:

(8) $${\text{SNR=20log}}_{10}\frac{\int_{f_{\max}+B/2}^{f_{\max}+B/2}\left|P_{x}(f)\right|df}{\int_{0}^{\infty }\left|P_{x}(f)\right|df-\int_{f_{\max}+B/2}^{f_{\max}+B/2}\left|P_{x}(f)\right|df}$$

Because of self-correlation, the SNR after processing is calculated as below:

(9) $${\text{SNR=10log}}_{10}\frac{\int_{f_{\max}+B/2}^{f_{\max}+B/2}\left|P_{x}(f)\right|df}{\int_{0}^{\infty }\left|P_{x}(f)\right|df-\int_{f_{\max}+B/2}^{f_{\max}+B/2}\left|P_{x}(f)\right|df}$$

where B = 0.05 Hz is the resolution in the estimation of the periodogram. SNR calculations are shown in Table 1. When there is a person in front of the radar, the average SNR improvement by the method described in this paper is 23.5 dB. When there is no person in front of the radar, there are still peaks in the frequency spectrum and the average SNR improvement by this paper’s method is 17.9 dB. After experiments in many different environments and with different human postures, we can conclude that, after processing, when the SNR is greater than 10 dB, it can be verified that the person in front of the radar has vital signs. In order to check the SNR improvement by ALE technique, this paper adopts a low pass FIR filter with 300 orders, Hamming window, and 1 Hz cut-off frequency to replace the ALE technique. The results are shown in Table 1. When there is a person in front of the radar, comparing the same orders of the ALE technique with the FIR method, the average SNR improvement by the ALE technique is about 5 dB bigger than with the FIR method. When there is no person in front of the radar, the average SNR improvement by the ALE technique is about 4 dB less than with the FIR method. Therefore we can conclude that to improve the vital sign recognition accuracy, the ALE technique is better than the FIR method.

**Table 1:** SNR of respiration signals in different experiments.

SNR	Person (3 m)	Person (5 m)	Person (7 m)	No Person
Original Signal	−7.82 dB	−8.13 dB	−18.31 dB	−19.21 dB
After Processing	12.39 dB	13.71 dB	10.08 dB	−1.33 dB
After Processing(FIR replacing ALE)	6.76 dB	11.27 dB	3.27 dB	2.76 dB

## 5. Conclusions

This paper decribes a new application of a 24 GHz respiration radar sensor and methods for non-contact detection of the vital signs of human subjects. A compact 24 GHz Doppler radar combined with suitable signal conditioning circuits can detect human respiration signals. In most of the previous studies, the continuous wave Doppler radar was used for detecting respiration signals of human subjects indoors, so the interferences caused by moving objects were relatively small, and the SNR was high. When the Doppler radar works outdoors, the interferences caused by various moving targets are very serious and common. In this case, the SNR of the radar echo signal was low, and it was difficult to extract respiration signals from the complicated background noise and moving object interference.

In order to minimize the interferences caused by moving objects, some methods were used for improving the SNR. Adaptive line enhancer [15], blind source separation [16], and empirical mode decomposition [17] were also used for extracting respiration signal from noise, but those methods were used when the SNR was relatively high. In order to avoid interferences caused by moving objects around the human subject outdoors, this paper uses signal processing methods involving baseline removal, self-correlation and adaptive line enhancement. Our experimental results show that the respiration signals can be extracted well with low SNR at a distance of 7 m. As future work smart vehicles equipped with cameras and the 24 GHz Dopple radar system will be tested together to search for simulated targets in a large area, so as to check the reliability of our proposed survivor searching system and the signal processing methods.
